# The antibacterial effectiveness of lavender essential oil against methicillin-resistant *Staphylococcus aureus*: a systematic review

**DOI:** 10.3389/fphar.2023.1306003

**Published:** 2023-12-07

**Authors:** Sandra Truong, Poonam Mudgil

**Affiliations:** School of Medicine, Western Sydney University, Campbelltown, NSW, Australia

**Keywords:** lavender essential oil, infectious diseases, methicillin resistant *Staphylococcus aureus*, MRSA, antibiotics, antimicrobial resistance

## Abstract

With the overuse and misuse of antibiotics, multi-drug resistant organisms have become a prominent issue in healthcare, increasing morbidity and mortality in affected patients. One such organism of concern is methicillin-resistant *Staphylococcus aureus* (MRSA) which is a leading cause of a variety of clinical infections. Therefore, in the interest of finding alternate substances to antibiotics, there has been increased interest in the antibacterial properties of lavender essential oil (LEO). This systematic review aims to collate information regarding the antibacterial properties of LEO against *S. aureus* and MRSA. A systematic search was conducted across four databases between the years 2002 and 2022, and through this, 23 studies were included in this paper. These studies used a variety of methods to ascertain the antibacterial effectiveness of LEO alone or in combination with other substances. Overall, there were mixed results regarding the antibacterial effectiveness of LEO against *S. aureus* and MRSA, with some studies reporting significant effectiveness, while other studies reporting a minimal to negligible effect. However, findings suggest that LEO works synergistically with other antibacterial substances, and it would be beneficial for additional research to be conducted in this area.

## 1 Introduction

Since their discovery, antibiotics have greatly increased our quality of life, treating infections which were once considered life-threatening. However, our reliance and increased antibiotic usage has contributed to the emergence of several antibiotic resistant bacterial strains.

As *Staphylococcus aureus* is a commensal mucosal organism in almost half the population, our repeated use of antibiotics has inevitably caused the development of multiple methicillin resistant *S. aureus* (MRSA) strains (Cameron et al., 2019; Nikolic et al., 2020). MRSA causes various clinical infections, being a leading cause of bacteraemia, endocarditis, skin and soft tissue infections, osteoarticular infections and device related infections (Tong et al., 2015; Ortwine and Bhavan, 2018). MRSA infections, relative to typical *S. aureus* infections, have increased mortality, increased hospitalisation rates and delayed access to sensitive antibacterial therapy (Delaney et al., 2008; Turnidge et al., 2009; Wehrhahn et al., 2010). This has resulted in increased hospital stay lengths, readmissions and poorer patient outcomes (Andreassen et al., 2017). To treat MRSA infections, clinicians have also often resorted to last line antibiotics such as vancomycin and daptomycin (Nikolic et al., 2020). While associated with a growing number of healthcare associated infections, there are recent concerns of MRSA also spreading throughout the Australian community (Tong et al., 2015; Cameron et al., 2019). This is especially concerning as MRSA infection rates have been increasing faster than healthcare utilisation rates (Nimmo et al., 2008). Therefore, to combat antibiotic resistance, it is imperative to find alternate antimicrobial substances. Thus, the antimicrobial activity of many traditional remedies for infections are currently being studied to evaluate their effectiveness (Dagli et al., 2015).

Essential oils (EO) are volatile and fragrant concentrated plant extracts used as alternate medical remedies since the 12th century (Man et al., 2019). These oils can be extracted from various part of the plant, including the leaves, roots, flowers, fruits, resin, seeds and bark (Man et al., 2019). Currently EOs are commonly used in a variety of products, including soaps, lotions, insect repellents, foods, fragrances, and laundry detergents (Ramsey et al., 2020). As an alternative medicine, EOs have also successfully been used to reduce postoperative nausea and an autonomic pain response, reducing pain associated with chronic conditions and medical procedures and symptomatic relief in cancer patients (Soltani et al., 2013; Kiberd et al., 2016; Bikmoradi et al., 2017; Ho et al., 2017; Mahboubi, 2017). However, with the development of modern chemistry, it has been noted that EOs contained various bioactive compounds unique to each plant, with antioxidant and antimicrobial potential (Man et al., 2019). This antimicrobial effect has also been reported on multidrug resistant strains (Soliman et al., 2017; Vasireddy et al., 2018).

Lavender essential oil (LEO), from the *Lamiceae* family, is a popular and common commercially available EO noted to possess antimicrobial properties (Cavanagh and Wilkinson, 2005). This is speculated to be because of its chemical compounds. Despite multiple studies testing the antibacterial effect LEO on *S. aureus* and MRSA, there has been no systematic review specifically conducted on this topic.

Thus, this systematic review primarily aims to collate and review data from the primary articles which have investigated the antimicrobial effects of lavender oil on *S. aureus* and MRSA. The secondary outcome of this systematic review involves the extent of LEO effectiveness against *S. aureus* and MRSA, as well as the potential difference of activity between LEO varieties and whether LEO, when used synergistically, can improve the antimicrobial effect of other substances.

## 2 Methods

A systematic review investigating the *in vitro* antimicrobial effectiveness of lavender oil on *S. aureus* and MRSA was conducted according to the Preferred Reporting Items for Systematic Reviews and Meta-analyses (PRISMA) guidelines. A database search of the registries such as the International Prospective Register of Systematic Reviews (PROSPERO); Joanna Briggs Systematic Review Register and Epistimonikos indicated that no systematic review had been conducted on this topic previously.

### 2.1 Research question

Does lavender oil used alone or in conjunction with other agents exhibit an effective antimicrobial effect against *S. aureus* and MRSA compared to with no intervention and with other bacteria?

### 2.2 PICO question

P (population): Against *S. aureus* and MRSA.

I (intervention): The addition of lavender oil.

C (comparison): Control/no treatment.

O (outcome): Effectiveness of antimicrobial properties.

### 2.3 Search strategy

The literature search was conducted on seventh of February 2022 in four databases: Embase, Web of Science, PubMed, and Medline. The search key words included (“lavender essential oil” or “lavender oil” or “lavandula”) AND (“antimicrobial” or “antibiotic” or “antibacterial”) AND (“staph *aureus*” or “*S. aureus*” or “*Staphylococcus aureus*”) AND (“MRSA” or “methicillin-resistant *S. aureus*” or “methicillin resistant *Staphylococcus aureus*”). The articles were imported into EndNote library.

### 2.4 Eligibility criteria

#### 2.4.1 Inclusion criteria

Articles published within the last 20 years were included, with the specific date ranges being 1 February 2002 to 31 January 2022. We included all types of methodology that assessed the antibacterial effectiveness of lavender oil against *S. aureus* and MRSA. This included various methods of vapour testing, broth microdilution, disc diffusion and wound dressing models. All varieties of lavender used to create lavender oil were also included. Articles which investigated lavender oil as the sole agent as well as lavender oil used in conjunction with another agent was also included. Articles which tested a range of essential oils were also included if they also tested the antimicrobial effectiveness of lavender oil.

#### 2.4.2 Exclusion criteria

Studies were excluded if they were not written in English and if there was no access to full text articles. Non-primary research articles, grey literature and opinion articles were also excluded. Texts where lavender oil was further processed to isolate a pure chemical compound were also excluded. Studies were also excluded if the substance tested was a lavender extract, and not an essential oil.

### 2.5 Study selection

After removing duplicates, title and abstract search was done and inclusion and exclusion criteria were applied to include relevant articles, then full text was searched to exclude articles that did not align with the inclusion criteria. The search selection was done independently by two reviewers (ST and PM) and conflicts were resolved by mutual consensus.

### 2.6 Study quality and risk of bias assessment

To ensure sound study quality, studies were chosen if they followed standard methods of microbial testing such as CLSI guidelines. There is no publicly available tool to assess *in vitro* studies of such variation.

### 2.7 Data extraction

Data was extracted from the included articles and organised in a table containing information on study location, intervention, methodology, objectives, and key findings.

### 2.8 Outcomes of interest

The primary outcome for this systematic review was to collate and evaluate available data on the antibacterial effectiveness of lavender oil on *S. aureus* and MRSA strains. Specifically, we focused on whether lavender oil had any antibacterial effect on *S. aureus*, with that being defined as inhibition of growth or a bactericidal effect. This included any studies that reported on a zone of inhibition produced on an inoculated agar plate as well as reports of an MIC or MBC.

Secondary outcomes included the extent of this antibacterial effect, the minimal concentrations required for this effect to be exhibited and whether the effectiveness was increased when lavender oil was used in conjunction with another compound.

## 3 Results

### 3.1 Search results

**FIGURE 1:**
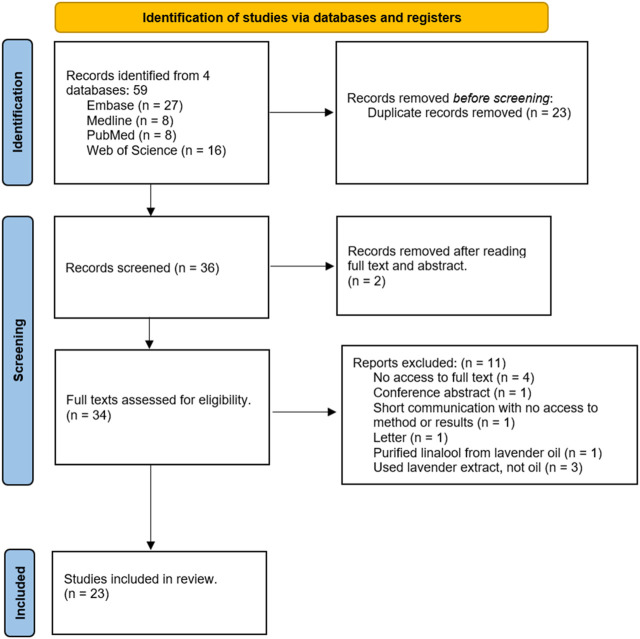
Preferred Reporting Items for Systematic Reviews and Meta-Analysis (PRISMA) flow diagram showing the study selection process.

The search netted a total of 59 results. The search process and reasons for study exclusion have been presented in the PRISMA flow chart below in Figure 1. After using EndNote to remove duplicates, there were a total of 36 articles. After reading the title and abstract, a total of 34 articles were found relevant to the research topic. One study was removed as the lavender oil was not tested on any *S. aureus* strain, and another was removed because a substance was created from lavender oil, meaning the oil itself was not tested. After screening full texts, 11 articles were excluded according to the diagram below, resulting in an inclusion of 23 articles.

### 3.2 Included studies

All studies were primarily laboratory studies focused on lavender oil as an antibacterial agent in the *in vitro* setting against *S. aureus* and MRSA. The extracted data from the included studies is presented in Table 1.

**TABLE 1:** The antibacterial effectiveness of lavender essential oil against methicillin-resistant *Staphylococcus aureus*: A systematic review (included studies and their key findings).

Author; year; country	Intervention	Methodology	Objective	Key findings
Abers et al., 2021; United States of America	**Substances:** Commercial LEO **Strains** MRSA and MSSA	Modified zone of inhibition assay according to CLSI	To evaluate the antimicrobial effectiveness of volatile substances emitted from EOs	Vapourised components of lavender oil had low antibacterial activity against *S aureus* and MRSA. (10–20 mm zone of inhibition)
Adaszynska-Skwirzynska et al., 2020; Poland	**Substances**: Commercial LEO (*L. angustifolia*) and gentamycin.	1. Chromatographic analysis on LEO.	To evaluate whether lavender oil exhibited antibacterial effects alone and synergistically with gentamycin	1. LEO had a very strong synergistic effect with gentamycin against MRSA and MSSA.
	**Strains**: *S. aureus* ATCC 25923 and clinically sourced MRSA	2. Microdilution checkerboard for MIC (CLSI).		2. LEO MICs: MSSA: 0.25% v/v, MRSA: 1.25% v/v
		3. FIC calculated from MIC.		3. LEO and gentamycin MIC: MSSA: 0.125 μg/ml, MRSA: 32.0 μg/ml
		4. Combination checkerboard for synergistic effects of LEO and gentamycin		
Bekka-Hadji et al., 2016; Algeria	**Substances**: LEO made by hydrodistillation (*L. stoechas*).	1. GC/MS analysis of LEO.	To evaluate the antibacterial activity of five Algerian medicinal plants from Lamiaceae family against MRSA	1. *T. fontanesii*, *O. glandulosum*, and *T. numidicus* were all more active than lavender oil.
	**Strains**. *S. aureus* ATCC 25923, MRSA ATCC 43300, MRSA S19	2. *In vitro* activity tested in aromatogram/disc diffusion assay.		2. Undiluted LEO: moderate activity against MSSA.
		3. MIC by broth microdilution (CLSI).		3. LEO anti-MRSA activity: similar to Roller et al.
		4. MBC by plating 100 µL from wells of LEO concentration equal or higher than MIC		4. Diameter of *L. stoechas* Microbial Inhibition zones (mm) for 100%, 50%, 25%, 12.5% and 6.25% oil. For *S. aureus* ATCC 25923: 17.7 ± 1.8; 11.7 ± 3.0; 08.6 ± 0.8; 08.0 ± 0.2; 07.4 ± 0.7. For MRSA strains: 16.4 ± 0.7 to 17.7 ± 1.2; 13.7 ± 0.3 to 14.4 ± 0.8; 09.1 ± 1.2 to 10.7 ± 0.6; 08.3 ± 1.3 to 08.7 ± 1.0; 06.3 ± 0.2 to 06.6 ± 0.3.
				5. LEO MIC and MBC (μL/ml): *S. aureus* ATCC 25923: 1.20 and 1.20; MRSA ATCC 43300: 4.70 and 9.40; MRSA S19: 0.30 and 1.20
Bona et al., 2019; Italy	**Substances:** LEO made using *L. latifolia* oil. **Strains:** 17 clinical MRSA strains, 27 clinical MSSA strains, *S. aureus* NCTC 6571	1. GC/MS analysis.	To test the inhibition efficacy of 12 different essential oils against clinical isolates of *S. aureus* including MRSA strains	1. No significant difference in efficacy of EOs between MRSA and MSSA.
		2. Disc diffusion assay (EUCAST).		2. LEO needed at higher concentration than other oils (oregano, winter savory, basil, and mint) for similar effects.
		3. MICs of EO with higher sensitivity than vancomycin calculated with microplate serial dilution MIC (EUCAST).		3. MIC % (v/v): Lavender oil effective against at least 50% of tested strains. MIC: 1–2 against most strains and 0.25 and 0.5 on one occasion.
		4. Fluorescein Diacetate Assay		4. Fluorescein Diacetate Assay: LEO reduced metabolic activity by at least 50% at maximum concentration (4%). Sub-MIC concentrations not effective at reducing metabolic activity
Brozyna et al., 2021; Poland	**Substances**: Commercial *L. angustifolia* LEO. **Strains**: *S. aureus* 6,538 and 33,591, Clinically isolated 6 strains of MSSA and 8 strains of MRSA	1. GC/MS analysis.	To test the antimicrobial and antibiofilm activity of essential oils in liquid and vapour phase against *S. aureus*	1. Vapour Phase: No inhibition of growth in most *staphylococcus* strains.
		2. Biofilm biomass level using crystal violet Assay.		2. Disc Diffusion: 9–12 mm inhibition zone when used in liquid form, 14 mm of partial inhibition in one case.
		3. Biofilm metabolic activity level using tetrazolium chloride staining.		3. MIC: MIC (v/v%) ranged from 0.2 to 3.1.
		4. Inverted Petri dish assay.		4. Biofilm: No antibiofilm activity observed in emulsified LEO.
		5. Disc diffusion assay.		5. Non-emulsified LEO - only slightly eradicated biofilm, but in some cases, it enhanced the biofilm.
		6. Serial microdilution for MIC.		6. LEO also had largest emulsion droplet size, which may have impacted its effectiveness as an antimicrobial agent. LEO droplet size: 3,531 ± 204 nm
		7. Minimal biofilm eradication assay.		
		8. Antibiofilm dressing activity.		
		9. AntiBioVol method.		
		10. Dynamic light scattering to measure EO emulsion droplet size		
Budzynska et al., 2011; Poland	**Substances**: Commercial LEO (*L. angustifolia*).	1. MIC by microdilution method with modified CLSI. LEO diluted with ethanol (1:1).	Examine the antibiofilm activity of select EOs (including LEO) and some of their major constituents	1. TTO, alpha-terpineol and terpinen-4-ol as well as MEO had stronger anti-biofilm activity than LEO.
	**Strains**:*S. aureus* ATCC 29213	2. TTC reduction assay to detect eradication of biofilm.		2. MBC was less than 4 x MIC. Against *S. aureus*, LEO had MIC: 0.78 %v/v, MBC: 1.56 %v/v, MBEC (4 h): 1.56 %v/v, and MBEC (24 h): 1.56 %v/v.
		3. Time dependent eradication of biofilm conducted on microplate.		3. Eradication of biofilm on surgical mesh: Needed 4-8x MIC to have >90% eradication of live bacteria.
		4. LIVE/DEAD BacLight Bacterial Viability kit to assess viability of cells treated with EOs		4. LEO has some antibiofilm potency
Cui et al., 2021; China	**Substances**: Commercial *L. spica* EO, combined lavender oil with erythromycin, streptomycin, ampicillin, florfenicol, chloramphenicol, tetracycline, gentamicin, kanamycin, bacitracin, amikacin and vancomycin.	1. Agar well diffusion test for oil alone.	To rapidly screen for the ability of 29 essential oils, including lavender oil, to enhance antibiotic activity	1. LEO MIC not noted as strong - 0.625 v/v%.
	**Strains**: MRSA 43300	2. Modified well diffusion method for antibiotic and oil.		2. Optimum concentration to reach acceptable zone of inhibition: 6.25 v/v %.
		3. MIC of essential oil with broth microdilution method (CLSI).		3. LEO had high-level enhancement with gentamycin on MRSA 43300.
		4. Modified well diffusion method for combination of antibiotic and essential oil.		4. Lavender oil not considered effective enough for time kill assay
		5. Time kill assay (not done on LEO)		
Di Vito et al., 2021; Italy	**Substances**:LEO and hydrolates made with *L. angustifolia* and *L. intermedia.*	1. GC/MS analysis and gravimetric analyses.	To compare the antimicrobial activity between essential oils and hydrolates from six Italian aromatic plants (including two lavender species). To compare the concentration of active volatiles in essential oils to hydrolates	1. While essential oils had a lower MIC, the volatiles in the hydrolates had higher antimicrobial effectiveness because they were active at lower concentrations. This is because they are hydrophilic and are more bioavailable than the essential oil.
	**Strains**: Clinical MRSA and MSSA strains	2. Microdilution broth to find MIC and minimum lethal concentration of EOs and hydrolates (EUCAST)		2. *Lavandula angustifolia* MIC and MBC % (v/v): MRSA: MIC: >2, MBC: >2; MSSA: MIC: >2, MBC: >2.
				3. *Lavandula intermedia* MIC and MBC % (v/v): MRSA: MIC: 2, MBC: >2; MSSA: MIC: 2, MBC: >2.
				4. Hydrolates from lavender species did not show antimicrobial effectiveness against MRSA or MSSA. 5. Of the oils tested, lavender had least effectiveness because of the least active chemicals
Edward-Jones et al., 2004; United Kingdom	**Substances**: Commercial LEO (*L. angustifolia*).	1. Oils loaded on filter paper discs placed on agar plates with *S. aureus.*	To determine the effect of essential oils on MRSA when used in a dressing model	1. Dressing Model: Effectiveness of EO depended on primary layer of dressing. Lavender oil showed some inhibition but wasn’t one of the most effective oils.
	**Strains**: 3 MRSA strains	2. Vapours assessed by putting the discs on the underside of the lid 8 mm away from agar surface.		2. LEO zone of inhibition: 15–21 mm.
		3. The most inhibitory combinations of oils were placed onto four layered dressing model		3. MRSA not susceptible to vapours from any essential oil. Lavender oil vapour had no antimicrobial effectiveness
Haba et al., 2014; Spain	**Substances**: Commercial *L. angustifolia* LEO. Rhamnolipids as an emulsifying agent.	1. Rhamnolipid production and characterisation with LC/MS.	To investigate whether essential oil composition influences emulsification with rhamnolipids and their use as antimicrobial agents against MRSA and *Candida albicans*	1. LEO MIC % (v/v) against MRSA: .
	**Strains**: MRSA ATCC 43300	2. Titration to determine the emulsifying properties of rhamnolipid.		2. Zone of Inhibition (LEO alone): No inhibition effect.
		3. Broth microdilution assay and resazurin assay to determine MIC of LEO and rhamnolipids.		3. Zone of Inhibition (LEO emulsion): The least effective emulsion was LEO (10.0 mm).
		4. Agar-well diffusion to assess antimicrobial activity of emulsions. Emulsions had a high EO content, within therapeutic range. LEO emulsions concentration: 78.7/8.5/12.8 (% water/rhamnolipid/EO)		4. The concentrations of LEO used are safe for topical use, but other EOs showed more effective inhibition
Kırmızıbekmez et al., 2009; Turkey	**Substances**: oil made from *L. stoechas.*	1. GC-FID and GC/MS analysis on oil.	To assess the chemical composition and antimicrobial activity of *L stoechas* that grow wild in Turkey	1. Flower essential oil was more active than the leaf oil towards the tested pathogenic microorganisms.
	**Strains**: Clinical strain of MRSA	2. Broth microdilution for MIC.		2. MRSA was more susceptible to LEO extracted from flowers (MIC = 31.2 μg/ml).
		3. TLC-bioautographic DPPH assay to detect free-radical scavenging activity of the samples		3. The most susceptible microorganism was MRSA, which when treated with the leaf and flower oils, produced MICs of 125 and 31.2 μg/ml, respectively
Koca et al., 2019; Turkey	**Substances**: *L. intermedia* oil.	Broth microdilutions to determine MIC (CLSI guidelines)	To investigate the antimicrobial effect of essential oils obtained from Mediterranean region plants on microorganisms isolated as secondary skin infections in patients with Grade II and III acute radiation dermatitis	1. *L. intermedia* MIC (µg/ml) against MRSA: 188, MSSA: 94, MRCNS: 188, and MSCNS: 47.
	**Strains**: Nine pathogenic strains isolated from 20 clinical samples. Pathogens isolated: MRSA, MSSA, MRCNS, MSCNS			2. *L. intermedia* was an effective antimicrobial against *S. aureus* isolated from acute radiation dermatitis
Kot et al., 2019; Poland	**Substances:** EO made from *L. angustifolia.*	1. GC-FID analysis on oil.	To examine the chemical compositions and antibacterial activity of EOs of five Lamiaceae species native to Poland to determine their potential for use against clinical MDR MRSA strains.	1. LEO activity against MDR MRSA was low efficiency.
	**Strains:** 18 strains of clinically isolated MDR MRSA	2. Two-fold serial dilution to determine MIC.		2. LEO MIC values for most strains were 3.12 mg/ml and for some strains 6.26–12.5 mg/ml.
		3. MIC values carried out by resazurin microtiter plate assay. No colour change indicated the MIC reached.		3. LEO MBC values for most strains were 4 MIC and 8 MIC (Ranging from 3.12 mg/ml to 100 mg/ml).
		4. MBC calculated by pipetting samples from wells that had MIC and incubating onto agar plates		4. Reason - this may be because this oil had fewer active compounds of linalool and linalyl acetate
Kwiatkowski et al., 2019; Poland	**Substances:** Commercial *L. angustifolia.*	1. Broth microdilution to determine MIC. Done according to CLSI standards with slight modifications.	To investigate the impact of LEO on OCT efficiency towards MRSA.	1. Both OCT and LEO showed antibacterial activity individually against MRSA clinical strains. LEO MIC: 13.72 ± 0 to 18.29 ± 7.92 mg/ml; OCT MIC: 1.95 ± 0.00 μg/ml to 3.91 ± 0.00 μg/ml; LEO MBC: 27.44–439 μg/ml.
	**Strains:** 4 strains of MRSA	2. MBC of LEO calculated by inoculating MIC wells.		2. LEO synergistically increased OCT’s susceptibility against MRSA strains and enhanced its effect as an antiseptic. LEO-OCT MIC: 1.29 ± 0.49 mg/ml (0.13 ± 0.05%).
		3. Checkerboard assay for synergistic effect of LEO and OCT.		3. FIC and FICI noted, LEO and OCT had synergistic effect against all MRSA strains.
		4. Time-Killing Curves.		4. Time-kill assay for MRSA strains showed synergy between LEO and OCT. LEO likely allowed more OCT to permeate into cell.
		5. FTIR Analysis.		5. FTIR analysis: There were cell wall modifications in MRSA strains cultured in media supplemented with OCT or LEO/OCT.
		6. Calculated FIC and FICI of OCT-LEO		6. EOs likely act on cytoplasmic membrane causing a loss of membrane stability and increased permeability
Man et al., 2019; Romania	**Substances:** Commercial *L. angustifolia* EO.	Two adapted broth microdilution methods (CLSI 2018) to account for hydrophobicity of oil.	To investigate the effect of some commonly used essential oils in micellar and aqueous extract on some of the most common pathogenic bacteria	1. Lavender oil had a very low MIC, but a very high MBC. This is because some components affected cell division. MIC % (v/v): Lavender MiEO: 3.1% MRSA, 3.1% MSSA; Lavender AqEO: 25% MSSA, 50% MRSA. MBC: not reached for LEO micelles or aqueous solutions.
	**Strains:** MSSA and MRSA	1. Made homogenous micelles of water and EO that could mix with water-based liquid culture medium.		2. Better results achieved with micelles than aqueous solution, and aqueous solutions were less effective than ethanol on all bacteria.
		2. The second method followed the antimicrobial activity of hydrosoluble components of EOs.		3. Micelles likely exhibited antibacterial activity due to high levels of linalyl-butyrate (26.5%), and the soluble linalool (25%) was the likely inhibitory agent in the aqueous extract.
		3. HPLC analysis to assess chemical content of EO.		4. Negligible differences noticed between LEO’s effects against MSSA and MRSA.
		4. Spot inoculation on checkerboard pattern of last 3 microplate wells from MIC to find MBC		5. MRSA was less susceptible to AqEO than MSSA.
				6. Gram-positive peptidoglycan cell wall allows hydrophobic molecules to penetrate and reach the internal environment.
				7. Hydrophobicity of EOs likely disrupted bacterial structures, degrading the cell wall and cytoplasmic membrane This caused cytoplasm coagulation and diffusion through the double lipid layer of the membrane, altering permeability and function
Mesic et al., 2021; Bosnia and Herzegovina	**Substance:** Commercial *L. angustifolia* EO.	1. *Allium cepa* assay to evaluate genotoxic potential of chemicals.	To investigate the cyto/genotoxic effects of lavender and immortelle EOs using plant cells (*Allium cepa*) and human lymphocytes, as well as their antimicrobial potential using nine strains of bacteria and fungi	1. LEO had cytotoxic and genotoxic effects on *A. cepa* root cells and in the lymphocyte assay.
	**Strains:** *S. aureus* ATCC 25923, MRSA ATCC 33591	2. Peripheral blood lymphocyte culture to test for potential mutagenic effects.		2. LEO exhibited very strong antimicrobial activity, and inhibited growth of all tested microbial strains in various degrees. (*p* ≤ 0.05).
		3. Disc diffusion assay according to National Committee for Clinical Laboratory Standards		3. LEO significantly inhibited growth of MDR MRSA in all tested concentrations.
				4. Higher antibacterial activity of LEO noted against Gram-positive bacteria, especially against *S. aureus.*
				5. Zone of inhibition (mm). For *S. aureus*: Pure EO: 46.17 ± 1.04 mm; 750 μg/ml EO: 46.50 ± 0.50 mm; 500 μg/ml EO: 31.50 ± 0.50 mm; 250 μg/ml EO: 33.50 ± 1.50 mm. For MRSA: Pure EO: 27.67 ± 2.52 mm; 750 μg/ml EO: 31.50 ± 0.50 mm; 500 μg/ml EO: 31.67 ± 4.16 mm; 250 μg/ml EO: 21.67 ± 1.53 mm.
				6. Antimicrobial activity acts on phospholipid layer of bacterial cell structure
Oancea et al., 2019; Romania	**Substances:** LEO made with *L. officinalis* and *L. angustifolia* (wild).	1. Hydrodistillation to make oil.	To investigate the physical and chemical makeup of 4 plant-source cosmetic waters and 7 EOs and evaluate their antibacterial properties	1. Wild lavender had more inhibition than regular lavender.
	**Strains:** MRSA	2. Disk diffusion assay		2. Zone of inhibition: *L. officinalis* EO–17 mm; Wild *L. angustifolia* EO–20 mm
Predoi et al., 2018; Romania	**Substances:** LEO made from *L. angustifolia.*	1. Dynamic light scattering measurements.	To investigate the direct, synergistic, and indirect antibacterial activities of lavender essential oil, basil essential oils and hydroxyapatite against different human pathogenic Gram-positive and Gram-negative strains	1. LEO had good inhibitory growth activity.
	**Strains:** MRSA 1144, *S. aureus* 1,426	2. GC/MS analysis on EO.		2. HapL material significantly enhanced antimicrobial activity when coated with low concentrations of LEO for all strains.
		3. Adapted diffusion method on agar plates to measure antimicrobial activity.		3. Inhibition zones: LEO: *S. aureus*: 25 ± 1mm, MRSA: 24 ± 0.5mm; HapL: *S. aureus*: 13 ± 2mm, MRSA: 10 ± 2 mm.
		4. Microdilution broth method to determine MIC and MBC.		4. MIC: LEO: *S. aureus*: 0.78% (v/v), MRSA: 0.78% (v/v); HapL: *S. aureus*: 0.31 mg/ml, MRSA: 0.31 mg/ml.
		5. Flow cytometry assay to detect antimicrobial action		5. MBC: LEO: *S. aureus*: 1.56%, MRSA: 1.56%. HapL: *S. aureus*: 0.62 mg/ml, MRSA: 0.62 mg/ml.
				6. Stated - Gram-positive bacteria has a thick layer of peptidoglycan that can inhibit the membrane-disrupting action of EO.
				7. Flow cytometry - LEO did not show strong depolarisation of bacterial membrane in MRSA or *S. aureus*
Ribeiro et al., 2020; Belgium	**Substances**: Commercial *L. stoechas* **Strains**: MSSA, 2 strains of MRSA	1. GC/MS Analysis. 2. Evaluated cytotoxic EO activity against human keratinocyte and fetal epithelial cell lines. 3. Direct activity tested with broth microdilution to find MIC (CLSI). 4. Synergistic activity between EOs and antibiotics tested with broth microdilution. Used to determine FIC and FICI. 5. LEO’s indirect activity tested with broth microdilution. EO at sub-MIC concentrations added with antibiotics. EO considered to have indirect effect if MIC. Sub-MIC concentration has been fixed at a non-cytotoxic concentration	To enhance or restore the activity of the antibiotic (on a resistant strain) without introducing a new active compound in the resistance equation	1. Direct ability: Spanish lavender had no antimibacterial effect against *S. aureus* strains. MIC was all >1,000 μg/ml.
				2. Indirect ability: Almost all combinations showed an additive or a synergistic effect against MRSA.
				3. Against MRSA, Spanish lavender decreased the MIC of penicillin V by 64-fold from 4 μg/ml to 0.06 μg/mL. As MIC of penicillin V against the MSSA was 0.015 μg/ml, LEO could potentially restore activity of penicillin V on MRSA to that of MSSA.
				4. Amoxicillin MIC was decreased by eight times when combined with Spanish lavender.
				5. Suggested that EOs are lipophobic and can penetrate and accumulate in the phospholipidic membrane.
				6. Synergistic activity: Spanish lavender only had additive effect when tested for synergistic activity
Roller et al., 2009; United Kingdom	**Substances:** Four commercial lavender oils (*L. angustifolia*. *L. latifolia*. *L. stoechas, and necrodane-rich L. luisieri*).	1. Testing vapour: Disc with oil was placed on underside of Petri dish lid in the centre.	To compare the antimicrobial efficacy of several lavender oils, used singly and in combination, on MSSA and MRSA	1. All 4 LEOs inhibited growth of MSSA and MRSA by direct contact, but not in the vapor phase. Inhibition zones 28–33 mm at oil 20 μL, increasing with dose.
	**Strains:** MSSA and clinical isolate of MRSA	2. Disc diffusion method.		2. Oils had similar zones irrespective of chemical composition of the oils or *S. aureus* strain.
		3. Vapour diffusion method		3. Most LEO combinations showed inhibition zones similar to those when oils used individually, but necrodane rich *L. luisieri* oil with *L. stoechas* or *L. langustifolia* produced inhibition zones that were two times bigger than when each oil used individually.
				4. Difference in MRSA & MSSA sensitivities were insignificant.
				5. Acknowledged that disc diffusion may be inaccurate for hydrophobic substances
Said et al., 2015; Portugal	**Substances:** EO made from *L. coronopifolia.*	1. GC/MS Analysis.	To analyse composition of EO of *L. coronopifolia* from Morocco and evaluate its *in vitro* antibacterial activity against antibiotic-resistant bacteria isolated from clinical infections	1. Clear bactericidal effect of *L. coronopifolia* EO against MRSA.
	**Strains:** Clinical MRSA	2. Broth microdilution for MIC and MBC.		2. MIC: 1% (v/v), MBC: 2%.
		3. Disc diffusion		3. LEO was effective against almost all bacteria studied, with the highest activity against MRSA, producing a 16 mm zone of inhibition
Voravuthikunchai et al., 2012; Thailand	**Substances:** EO made from *L. angustifolia* EO.	1. Hydrodistillation to extract EO.	To check the effectiveness of selected EOs against different pathogenic bacteria in solid phase by disk diffusion; to evaluate their effectiveness in vapour phase; and to apply the atmosphere generated by the most effective EOs in a portable air conditioning prototype	1. LEO showed moderate inhibitory effect in agar disc diffusion.
	**Strains:** 11 clinical isolates of MRSA	2. Paper disc agar diffusion (CLSI).		2. For LEO - Zone of inhibition: *S. aureus* ATCC 25923: 18 ± 1mm, MRSA: 12 ± 1mm; MIC: *S. aureus* ATCC 25923: 1:16, MRSA 1:8; MBC: *S. aureus* ATCC 25923 1:8, MRSA 1:8; Vapour contact inhibition zones: *S. aureus*: 18 ± 1mm, MRSA: 12 ± 1 mm.
		3. Vapour diffusion assay.		3. For oil blend containing 23% LEO - Inhibition zones: *S. aureus* ATCC 25923: 28 ± 1mm, MRSA: 25 ± 1mm; MIC: *S. aureus* ATCC 25923: 1:16, MRSA: 1:16; MBC: *S. aureus* ATCC 2593: 1:16, MRSA: 1:32; also reduced the CFU of *S. aureus* and MRSA in the air-conditioning model within 1st hour: *S. aureus* 125 to 75 CFU, MRSA 125 to 100 CFU
		4. Modified agar microdilution method to determine MIC of EOs that produced inhibition zones.		
		5. Agar dilution method on EOs with significant efficacy to determine MBC.		
		6. Airconditioning model: EO was placed into closed aircon system. Inoculated Petri dish placed in aircon environment for 8-h intervals up to 24 h. Mean colony counts recorded		
Warnke et al., 2009	**Substances:** Commercial LEO.	Agar diffusion test	To evaluate the antibacterial and antimycotic efficacy of different EOs on frequently isolated and hospital-acquired bacterial strains including MRSA	1. LEO had antibactbial effect on all *S. aureus* strains.
	**Strains:** *S. aureus* ATCC 25923, *S. aureus* VA 10465/02, MRSA			2. LEO Zone of inhibition: *S. aureus* ATCC25923: 10mm, *S. aureus* VA 10465/02: 11mm, MRSA VA 10492/02: 12 mm
				3. Controls: ethanol (70%): 9mm, Povidone: 14mm, H_2_O_2_: 15mm, Chlorhexidine: 16mm, Olive oil: 0mm, Paraffin oil: 0 mm.
				4. EOs often diluted with ethanol, so used ethanol as control, suggested - if EO’s zone of inhibition higher than ethanol, the antibacterial effect was due to EOs.
				5. Effect not just because of oil nature, as olive oil and paraffin oil had no inhibitory effect. 6. Size of zone depended on solubility, so size was not direct indicator of antimicrobial effectiveness, instead indicated presence of antimicrobial effect

Abbreviations: LEO, Lavender essential oil; MRSA, methicillin-resistant *Staphylococcus aureus*; MSSA, methicillin-sensitive *Staphylococcus aureus*; CLSI, Clinical and laboratory standards institute; MIC, minimal inhibition concentration; FIC, fractional inhibition concentration; ATCC, American Type Culture Collection, GC/MS, analysis–gas chromatography/mass spectrometry analysis, LC/MS, liquid chromatography/mass spectrometry, MBC, minimal bactericidal concentration; EUCAST, European Committee on Antimicrobial Susceptibility Testing, AntiBioVol - antibiofilm activity of volatile compounds, EO, Essential Oil; TTC, 2,3,5-triphenyltetrazolium chloride, TTO, Tea tree oil; MEO, Melissa essential oil; MLC, minimal lethal concentration; GC-FID, gas chromatography flame ionisation detection, TLC-bioautographic DPPH, assay - Thin Layer Chromatography bioautographic 2,2-diphenyl-1-picrylhydrazyl assay, MRCNS, methicillin-resistant coagulase negative *Staphylococcus*; MSCNS, methicillin sensitive coagulase negative *Staphyloccocus*, MDR MRSA, multidrug resistant MRSA, OCT, octenidine; HPLC, analysis - High-performance liquid chromatography analysis, MiEO, micelle solution of essential oil, AqEO, aqueous phase of essential oil, HapL –hydroxyapatite coated with lavender essential oil, FTIR, analysis - Fourier Transform Infrared Spectroscopy analysis; FICI, Fractional inhibitory concentration indices; CFU, colony forming units; OCT-LEO, octenidine and lavender essential oil.

These studies were conducted across a wide range of countries. Many were conducted in Poland (Budzynska et al., 2011; Kot et al., 2019; Kwiatkowski et al., 2019; Adaszynska-Skwirzynska et al., 2020; Brozyna et al., 2021). Others were conducted in United States, Jordan, Algeria, Italy, China, UK, Spain, Turkey, Romania, Bosnia and Herzegovina, Belgium, Portugal, Australia and Morocco (Edwards-Jones et al., 2004; Kirmizibekmez et al., 2009; Roller et al., 2009; Warnke et al., 2009; Voravuthikunchai et al., 2012; Haba et al., 2014; Said et al., 2015; Bekka-Hadji et al., 2016; Predoi et al., 2018; Bona et al., 2019; Koca et al., 2019; Man et al., 2019; Oancea et al., 2019; Ribeiro et al., 2020; Abers et al., 2021; Cui et al., 2021; Di Vito et al., 2021; Mesic et al., 2021).

Overall, 16 methods of testing the antibacterial effect of lavender oil were used. The most common method of testing was microdilution, which was conducted by 15 studies. (Kirmizibekmez et al., 2009; Budzynska et al., 2011; Haba et al., 2014; Said et al., 2015; Bekka-Hadji et al., 2016; Predoi et al., 2018; Bona et al., 2019; Koca et al., 2019; Kwiatkowski et al., 2019; Man et al., 2019; Adaszynska-Skwirzynska et al., 2020; Ribeiro et al., 2020; Brozyna et al., 2021; Cui et al., 2021; Di Vito et al., 2021). This was followed by disc diffusion, conducted by 12 studies. (Edwards-Jones et al., 2004; Roller et al., 2009; Warnke et al., 2009; Voravuthikunchai et al., 2012; Said et al., 2015; Bekka-Hadji et al., 2016; Bona et al., 2019; Oancea et al., 2019; Brozyna et al., 2021; Mesic et al., 2021). Five studies were interested in the antimicrobial activity of LEO vapours (Edwards-Jones et al., 2004; Roller et al., 2009; Voravuthikunchai et al., 2012; Abers et al., 2021; Brozyna et al., 2021). Two studies also concerned themselves with LEO activity against *S. aureus* biofilms (Budzynska et al., 2011; Brozyna et al., 2021).

Due to the variety of experimental methods used, results on the antibacterial effectiveness were measured in different formats. Most commonly, the contents of microdilutions, which reported minimum inhibitory concentrations (MIC), were further spot inoculated to then determine a minimum bactericidal concentration (MBC). These concentrations were often measured in %v/v and µg/mL. Disc diffusions were also commonly given a zone of inhibition measured in mm. A summary of the methods used in each study can also be seen in the table of included studies. (Table 1).

Studies which tested the antibacterial effect of lavender oil vapours used modified versions of agar diffusion and independently designed methods, such as the air conditioner model ((Edwards-Jones et al., 2004; Voravuthikunchai et al., 2012; Abers et al., 2021; Brozyna et al., 2021).

### 3.3 Types of lavender oil

Some studies sourced their LEO commercially, while others extracted their own oil. Therefore many varieties of lavender were used, with the most common being *Lavandula angustifolia* (17 studies) (Edwards-Jones et al., 2004; Roller et al., 2009; Budzynska et al., 2011; Voravuthikunchai et al., 2012; Haba et al., 2014; Predoi et al., 2018; Kot et al., 2019; Kwiatkowski et al., 2019; Man et al., 2019; Oancea et al., 2019; Adaszynska-Skwirzynska et al., 2020; Abers et al., 2021; Brozyna et al., 2021; Di Vito et al., 2021; Mesic et al., 2021). Other varieties of lavender tested included *L. stoechas*, *L. latifolia, L. spica, L. intermedia, L. luisieri, L. coronopifolia, L. dentata* and wild *L. angustifolia* (Kirmizibekmez et al., 2009; Roller et al., 2009; Said et al., 2015; Bekka-Hadji et al., 2016; Bona et al., 2019; Koca et al., 2019; Oancea et al., 2019; Ribeiro et al., 2020; Cui et al., 2021). Warnke et al.‘s LEO was not specified (Warnke et al., 2009). Three studies tested multiple LEO varieties, comparing their antibacterial activities to each other (Roller et al., 2009; Oancea et al., 2019; Di Vito et al., 2021).

### 3.4 Strains of *S. aureus* and MRSA

*S. aureus* and MRSA strains were mostly clinically sourced and sourced from culture collections. The strains tested were *S. aureus* ATCC 14775, MRSA ATCC BAA-44, *S. aureus* ATCC 25923, MRSA ATCC43300, MRSA S19, *S. aureus* ATCC 6538, *S. aureus* ATCC 33591, Oxford MRSA NCTC 6571, EMRSA 15, methicillin sensitive *Staphylococcus aureus* (MSSA) ATCC 29213, MRSA ATCC 33591, MRSA 1144, *S. aureus* 1426, MSSA LMG 8064, MRSA LMG 15975, MRSA LMG 16217, MSSA - NCTC 6571, *S. aureus* VA 10465/02 and *S. aureus* VA 10492/02 MRSA. Additionally, there were also 73 strains of clinically extracted MRSA and 23 strains of clinically extracted MSSA (Kirmizibekmez et al., 2009; Roller et al., 2009; Voravuthikunchai et al., 2012; Said et al., 2015; Bekka-Hadji et al., 2016; Bona et al., 2019; Kot et al., 2019; Kwiatkowski et al., 2019; Adaszynska-Skwirzynska et al., 2020; Brozyna et al., 2021). Koca et al. also used methicillin-resistant coagulase negative *S. aureus* (MRCNS) and methicillin-sensitive coagulase-negative *S. aureus* (MSCNS) (Koca et al., 2019).

### 3.5 Primary outcomes

#### 3.5.1 Antimicrobial effectiveness of liquid lavender oil alone

Exact results from studies using disc diffusion and microdilution on LEO alone can be found on the table of included studies.

##### 3.5.1.1 Disc diffusion

12 studies used disc diffusion to assess the effectiveness of LEO on methicillin sensitive *S aureus* (MSSA) and MRSA. Voravuthikunchai et al. (2012) followed Clinical and Laboratory Standards Institute (CLSI) guidelines. Bona et al. (2019) followed European Committee on Antimicrobial Susceptibility Testing (EUCAST). One study followed the National Committee for Clinical Laboratory Standards (NCCLS) (Mesic et al., 2021). The remaining studies conducted standard disc diffusion, where EO or a mix of EO and solvent was dissolved then placed onto filter paper discs.

Predoi et al. (2018) used 5 µL of LEO and dimethylsulfoxide (DMSO) mix on each disc (Predoi et al., 2018). Nine studies used 10 µL of LEO on each disc (Edwards-Jones et al., 2004; Roller et al., 2009; Warnke et al., 2009; Voravuthikunchai et al., 2012; Said et al., 2015; Bekka-Hadji et al., 2016; Bona et al., 2019; Brozyna et al., 2021; Mesic et al., 2021). The discs were loaded onto inoculated agar plates and incubated at 37°C for 24 h. Warnke et al. (2009) incubated their strains for 18 h. Oancea et al. (2019) did not specify their exact disc diffusion methods. Overall, most studies had zones of inhibition ranging from 9 to 46 mm (Edwards-Jones et al., 2004; Warnke et al., 2009; Voravuthikunchai et al., 2012; Bekka-Hadji et al., 2016; Predoi et al., 2018; Bona et al., 2019; Oancea et al., 2019; Abers et al., 2021; Brozyna et al., 2021; Mesic et al., 2021). Haba et al. (2014) reported no inhibition by LEO on its own.

In studies which impregnated their discs with increasing amounts of LEO, it appears the zones of inhibition increased with the amount and concentration of LEO added to each disc (Bekka-Hadji et al., Roller et al., 2009). The one slight exception showed in Mesic et al.’s (2021) study, where the 75% concentration LEO showed a slight increase in zone of inhibition compared to LEO at full concentration (Mesic et al., 2021). Further dilutions then showed a decrease in inhibition zones (Mesic et al., 2021).

The inhibition zones across studies that used a set aliquot of LEO were varied. A common aliquot used across multiple studies was 10 μL of pure LEO, and the zones of inhibition yielded varied, from 10 to 46 mm (Roller et al., 2009; Warnke et al., 2009; Bekka-Hadji et al., 2016; Bona et al., 2019; Mesic et al., 2021). Warnke showed had zones from 10 to 11 mm. (Warnke et al., 2009). Bekka, Bona, Said and Roller had zones ranging from 16 to 23 mm (Roller et al., 2009; Said et al., 2015; Bekka-Hadji et al., 2016; Bona et al., 2019). Mesic was an outlier, with zones of inhibition of 27 mm for MRSA and 46 mm for MSSA, and there were no notable differences in their method of disc diffusion (Mesic et al., 2021).

Some studies used higher amounts of LEO, and these all produced varying zones of inhibition, some which were lower than studies who used only 10 μL. Between studies, it also showed that using a higher amount of LEO did not show a trend of increasing zones of inhibition. Brozyna et al. (2021) produced a max zone of inhibition of 14 mm using 200 μL and Edwards-Jones et al. (2004), produced a zone of inhibition of 20 mm with 20 μL (Edward-Jones et al., 2004; Brozyna et al., 2021). Haba et al. (2014) used 50 μL of LEO and showed no inhibition (Haba et al., 2014). Voravuthikunchai et al. soaked their paper disc in 10 ml of LEO before applying it to their agar plates, and therefore the exact amount of LEO on the paper disc is unknown (Voravuthikunchai et al., 2012). They produced zones of inhibition of 18 mm for MSSA and 12 mm for MRSA, which differs little to the zones produced by studies who used 10 μL (Voravuthikunchai et al., 2012).

Haba et al. (2014) and Predoi et al. (2018) also did studies mixing their LEO with solvents (Haba et al., 2014; Predoi et al., 2018). Predoi et al. mixed their LEO with a 50:50 ratio of LEO and DMSO (Predoi et al., 2018). Their disc, inoculated with 5 μL produced a zone of inhibition of 25 mm for MSSA and 24 mm for MRSA and the DMSO control showed no inhibition zone (Predoi et al., 2018). Haba et al. (2014), when emulsifying LEO with rhamnolipids and applying 50μL, created a zone of 10 mm inhibition (Haba et al., 2014). However, it is noted that the rhamnolipids themselves produced a 9 mm inhibition zone (Haba et al., 2014). Therefore, there is a possibility that solvents can increase the effectiveness of LEO against *S. aureus* in disc diffusion assays.

Three studies followed established guidelines set by CLSI, EUCAST and NCCLS (Voravuthikunchai et al., 2012; Bona et al., 2019; Mesic et al., 2021). These guidelines specified the methods, such as temperature and hours of incubation, the media required to grow MSSA and MRSA and which antibiotic controls and concentrations. The guidelines did not specify the amount of LEO that could be added to the disc.

The three studies that followed these guidelines used the recommended antimicrobials as controls at the required concentrations. Whilst Voravuthikunchai et al. (2012) stated strains were tested against amikacin, ampicillin, gentamicin, kanamycin and tetracyline, no comments were made regarding comparisons between LEO and these antibiotics (Voravuthikunchai et al., 2012). Bona et al. (2019) compared results to those of vancomycin, and showed LEO had a higher zone of inhibition than vancomycin, which they deemed a significant result (Bona et al., 2019). Mesic et al. (2021) compared their LEO results to ampicillin and deemed LEO inhibition to be significant compared to this antibiotic (Mesic et al., 2021).

##### 3.5.1.2 Microdilution

15 studies used microdilution to determine the MIC of LEO against *S. aureus*. Different studies expressed their MIC with different units, including % (v/v), µg/mL, μL/mL, mg/mL. The results expressed in % (v/v) had the MIC ranges of 0.2–12.5 (Budzynska et al., 2011; Voravuthikunchai et al., 2012; Haba et al., 2014; Said et al., 2015; Predoi et al., 2018; Bona et al., 2019; Adaszynska-Skwirzynska et al., 2020; Brozyna et al., 2021; Cui et al., 2021; Di Vito et al., 2021). Bekka-Hadji et al. (2016) expressed their MIC in μL/mL, and had MIC results ranging from 0.3–4.70. When converted to a %v/v MIC, this ranged from 0.03 to 0.47. Studies which expressed their MIC as weight per mL had their results range from 31.2 to 125 mg/ml (Kirmizibekmez et al., 2009; Koca et al., 2019; Kot et al., 2019; Kwiatkowski et al., 2019) Ribeiro et al.’s (2020) study however stated that their Spanish LEO had no antibacterial effect against their strains of MRSA and MSSA, which was an exception to the other studies. They reported their MIC as being >1,000 μg/ml.

Likewise MBC was also expressed in different units, including μL/mL, %v/v and mg/mL. Results reported in %v/v ranged from 1.56 to 12.5 (Budzynska et al., 2011; Voravuthikunchai et al., 2012; Said et al., 2015; Predoi et al., 2018; Di Vito et al., 2021). Results recorded in μL/mL ranged from 1.20 to 9.40 (Bekka-Hadji et al., 2016). When converted to %v/v. This ranged from 0.12 to 0.94. Finally, MBCs reported in weight per mL ranged from 27.44 to 100 mg/ml (Kot et al., 2019; Kwiatkowski et al., 2019). In Man et al. (2019) study, both aqueous and micellular forms of the LEO did not reach MBC.

Eight studies followed Clinical and Laboratory Standards Institute (CLSI) guidelines (Budzynska et al., 2011; Bekka-Hadji et al., 2016; Koca et al., 2019; Kwiatkowski et al., 2019; Man et al., 2019; Adaszynska-Skwirzynska et al., 2020; Ribeiro et al., 2020; Cui et al., 2021). Kwiatkowski et al. (2019) however made some slight modifications to these guidelines as per their previous study, where a final concentration of 1.0% (v/v) Tween^®^ 80 was added to the medium to enhance EO solubility (Kwiatkowski et al., 2018; Kwiatkowski et al., 2019). Budzynska et al. (2011) also made modifications to the CLSI guidelines by dilution LEO with ethanol at a 1:1 ratio. Two studies followed the European Committee on Antimicrobial Susceptibility Testing (EUCAST) (Bona et al., 2019; Di Vito et al., 2021).

##### 3.5.1.3 Other methods of testing lavender oil

Nine studies used other methods of testing lavender oil efficacy against MSSA and MRSA in alone in its liquid form. Bona et al. (2019) used a fluorescein diacetate assay to observe the metabolic activity of MRSA after exposure to varying concentrations of EO. LEO reduced MRSA metabolic activity by 50% at maximum concentration (4%), but metabolic activity quickly became unchanged once LEO was at sub-MIC concentrations (Bona et al., 2019).

Brozyna et al. (2021) recorded an antibiofilm dressing activity measurement following the antibiofilm activity of volatile compounds (AntiBioVol) protocol which showed that emulsified LEO had no effect on *S. aureus* biofilms. Additionally, non-emulsified LEO only slightly eradicated biofilm, and in some cases, enhanced it (Brozyna et al., 2021). Budzynska et al. (2011) also measured biofilm eradication across time and concentration using the TTC and MTT reduction assays. The assays revealed that LEO needed to be at 4–8 times the MIC in order to eradicate 90% of the biofilm (Budzynska et al., 2011).

Cui et al. (2021) used a modified well diffusion assay where oils were added in a range of 5,120 to 20 μg/ml until acceptable inhibition zones were produced (11–18 mm). In this assay, LEO was noted to have weak antibacterial effectiveness as it required a concentration of 6.25 v/v % to achieve an acceptable diameter.

Kot et al. (2019) performed a resazurin microtitre plate assay, where MIC values were determined when wells had no colour change. LEO had low efficacy antimicrobial effects against MRSA, with MIC values ranging from 3.12 to 12.5 mg/ml, and MBC values being four MIC to eight MIC for most strains.

Kwiatkowski et al. (2019) performed a time-kill assay on sub-MIC concentrations of LEO which showed little antimicrobial activity. However, this was done as a control for their subsequent LEO and octenidine time killing assay.

Predoi et al. (2018) used oxonol DiBAC4 as an indicator in flow cytometry assay to determine whether LEO could disrupt bacterial membrane potential at ½ x MIC. The fluorescence intensity in cells treated with LEO was less than that of ½ x MIC of DMSO in both MRSA and MSSA.

##### 3.5.1.4 Lavender oil vapours

Brozyna et al. (2021), Edward-Jones et al. (2004) and Roller et al. (2009) did not observe LEO vapours providing antimicrobial activity against MSSA or MRSA. Aber et al. (2021) observed low amounts of inhibition against MRSA and MSSA, however, this only occurred at the two highest doses of LEO (80–160 μL). Voravuthikunchai et al. (2012)’s vapour study observed LEO causing a zone of inhibition (MSSA: 18 ± 1 mm,MRSA: 12 ± 1 mm). However, unlike Aber et al. (2021) study, they did not specify a criteria to judge whether the presence of this zone showed significant antimicrobial activity. When using the air-conditioning model, Voravuthikunchai et al. (2012) also observed a reduction in colony forming unit (CFU) when exposing MSSA to a blend of oils (Cinnamon 23%, Lavender 23%, Lemon thyme 39%, Thyme 15%). Voravuthikunchai et al. saw a reduction of 300 to 100 CFU in the first hour, and then total eradication at 3 h.

#### 3.5.2 Antimicrobial effectiveness of lavender oil when used synergistically

Eight studies also tested lavender oil antibacterial activity in conjunction with other substances. Adaszynska-Skwirzynska et al. (2020) tested LEO with gentamycin. Cui et al. (2021) tested LEO with a range of antibiotics (erythromycin, streptomycin, ampicillin, florfenicol, chloramphenicol, tetracycline, gentamicin, kanamycin, bacitracin, amikacin, vancomycin) in a modified well diffusion method. Edward-Jones et al. (2004) used a four layer dressing model where 100 µL of EO combinations (LEO and geranium EO, LEO and citricidal EO, LEO and tea-tree oil) where placed onto the centre of gauze. The gauze was inoculated with *S. aureus*, covered with four layers of dressings and incubated (37°C for 24 h). The primary layer was modified with different combinations of FlamazineTM, Telfa ClearTM and JelonetTM. (Edwards-Jones et al., 2004). Haba et al. (2014) tested LEO with rhamnolipids in a well-diffusion assay. Kwiatkowski et al. (2019) tested LEO with octenidine (OCT) with microdilutions and a time-kill assay. The time-kill assay was performed by inoculating media containing LEO and OCT, incubating it at 37OC. 100 µL samples were removed at timepoints (0, 1, 2, 3, 4, 5, 6 12, and 24 h), serially diluted, spread onto Mueller-Hinton plates and incubated at 37°C to determine the mean colony counts (Kwiatkowski et al., 2019). Predoi et al. (2018) coated hydroxyapatite (Hap) with LEO and assessed the antimicrobial activity with microdilution. Ribeiro et al. (2020) tested LEO with penicillin and amoxicillin in a microdilution method. Roller et al. (2009) tested different varieties of LEO together. Voravuthikunchai et al. (2012) tested a blend of EOs in an independently designed air-conditioning model.

## 4 Discussion

With rising antibiotic use, the rise of antibiotic resistant organisms like MRSA has resulted in the need to broaden our current range of antibacterial agents. The surge of interest in essential oils and its use as a traditional medicine in some cultures has meant researchers have begun to conduct *in vitro* studies to investigate the effectiveness of EOs for a wide variety of purposes. Recent studies have shown that a variety of EOs, including LEO, have had promise as an antimicrobial agent. Overall, this systematic review revealed that MIC and MBC of lavender oil tended to vary across different studies. Overall, lavender oil was not effective as an antibacterial agent when used in its volatile state. Some studies stated that its lone use is effective against different strains of *S. aureus*, and others stating that the MIC required was very high before a significant effect was observed. Furthermore, some studies found that lavender oil had a positive synergistic effect when used with other agents.

### 4.1 LEO as the sole antibacterial agent against *S. aureus*

Results investigating LEO alone as an antibacterial agent against *S. aureus* were mixed. Studies have attributed this to the presence of various bioactive chemical components within the oil which itself have antibacterial properties. LEO’s hydrophobic nature could be responsible allowing it to incorporate within the bacterial membrane, weakening it, and allowing the bioactive components to enter the cell (Warnke et al., 2009; Bona et al., 2019; Man et al., 2019; Ribeiro et al., 2020). However, by additionally using olive oil and paraffin oil as a control it appears that the overall antibacterial effect of LEO cannot be singularly attributed to its lipophilic nature (Warnke et al. (2009). When testing LEO alone, antibacterial effectiveness was often measured in MIC, MBC and inhibition zones. Upon comparing these results between studies, it is noted that while LEO displayed antibacterial activity against MRSA and MSSA in most studies, the efficacy of this activity varied from study to study.

#### 4.1.1 MIC and MBC

MIC was a popular way to measure the antibacterial effect of LEO against *S. aureus*. Microdilution was often used to calculate an MIC. However, there is a large range of variability in results (0.03–12.5 %v/v and 31.2 μg/ml to 125 mg/ml) and they are hard to compare due to difference in units (Kirmizibekmez et al., 2009; Budzynska et al., 2011; Haba et al., 2014; Said et al., 2015; Bekka-Hadji et al., 2016; Predoi et al., 2018; Bona et al., 2019; Koca et al., 2019; Kot et al., 2019; Kwiatkowski et al., 2019; Adaszynska-Skwirzynska et al., 2020; Brozyna et al., 2021; Cui et al., 2021; Di Vito et al., 2021), with some cases showing no MIC (Ribeiro et al., 2020). Even when one looks exclusively at results from studies which followed CLSI and EUCAST guidelines, results are expressed in different units and have a wide range of variation (0.03–2%v/v and 13.72 mg/ml to nil). Some explanation for the variation between results was provided by creating aqueous and micellar solution of LEO (Man et al., 2019) but largely variations are difficult to compare. MBC likewise is also reported in different units and with a large range of variability (0.12–12.5%v/v and 27.44 μg/ml to 100 mg/ml) making it difficult to compare between studies (Budzynska et al., 2011; Voravuthikunchai et al., 2012; Said et al., 2015; Bekka-Hadji et al., 2016; Predoi et al., 2018; Kot et al., 2019; Kwiatkowski et al., 2019; Di Vito et al., 2021).

#### 4.1.2 Zones of inhibition

Disc diffusion was often implemented to observe the presence of antimicrobial activity, where most studies observed a zone of inhibition. Disc diffusion was used in 12 studies and results varied a lot (9–43 mm). When exclusively observing studies which used CLSI and EUCAST guidelines, though a narrower range was found (10–20 mm) but assay as per NCCLS guidelines had results on the higher side (27–46 mm) (Mesic et al., 2021) and there was also no zone of inhibition in another case (Haba et al., 2014). Therefore, while it appears LEO usually has antimicrobial effects against MRSA and MSSA, there is large variation between the effectiveness of its antibacterial properties between studies.

#### 4.1.3 Reasons for varied results

These inconsistent results between publications may have been caused by the slight variation of materials and methodology used by each study. Studies collected LEO from multiple varieties sourced from a range of countries. Some studies also extracted their own LEO, whilst others tested commercially available oils. Additionally, LEO was tested against various strains of *S. aureus*, all sourced from a variety of countries, collections, and clinical settings. Additionally, while some methods adhered to CLSI or EUCAST guidelines, other studies used standard methodology they had used previously in other studies which also tested the antimicrobial effect of other essential oils. While the presence of an MIC and MBC does indicate antibacterial activity of LEO against *S. aureus*, multiple studies did not focus on whether the antibacterial activity was enough for clinical applications. It is also difficult to compare the MIC and MBC between studies due to the variation in units. Had the methodology and materials been standardised, it would have been easier to compare results between studies or to identify whether a specific methodology was effective at testing LEO against *S. aureus*. The issue of hydrophobicity of oils may have also contributed to the inconsistency of MIC and MBC, as these measurements are typically reliant on the even dispersal provided by solubility. To address this, Man et al. (2019) attempted to create soluble aqueous and micellar solutions. The alteration of LEO likely contributed to the higher MIC values in their aqueous solution (25%–50%), but their micellar solution achieved a relatively low MIC within the range of the other studies (3.1%). (11) However, MBC was not achieved for either solution. Therefore, to improve the consistency of MIC and MBC values, it may be worthwhile to further consider solutions for dissolving LEO.

Additionally, the disc diffusion method could result in inconsistent findings as they are inaccurate when assessing substances which are insoluble such as oils. This is especially pertinent to consider as some studies added a solvent to encourage diffusion, whilst other studies did not. This has been acknowledged in many of the studies that have used this method. The insoluble nature of LEO may have also contributed to the varied results in studies using microdilution. Man et al. (2019) attempted to circumvent this by creating micelles and also by extracting the aqueous layer formed by LEO and water, and they identified LEO as being an active oil against MRSA and MSSA. Therefore, it would be promising if this exact methodology was repeated by another team and the results remained consistent and similar to Man et al.‘s findings.

#### 4.1.4 LEO against biofilm

There have also been mixed results regarding the effectiveness of LEO against *S. aureus* biofilm inhibition. No antibiofilm activity was reported in LEO emulsified with Tween 20 and some amounts of antibiofilm activity was found in non-emulsified LEO (40%–70% eradication) (Brozyna et al., 2021). While other EOs were stronger antibiofilm agents, LEO still had some antibiofilm potency (Budzynska et al., 2011). Overall it is difficult to ascertain the effectiveness of LEO against *S. aureus* biofilms as there are only two studies which have investigated this. Each study also used different methods of intervention, LEO and *S. aureus* strains (as explained below). The assessment of biofilm was also different between studies. This is likely because there have been various published methods of assessing biofilms (Sahra, 2019).

Brozyna et al. (2021) used crystal violet staining, another popular method of biofilm determination, to assess total biofilm mass, and its activity level was assessed with a tetrazolium chloride assay (TTC staining). Then, Brozyna et al. used a minimal biofilm eradication concentration (MBEC) assay to assess the ability of liquid LEO to eradicate biofilm with Tween 20 as the emulsifier. To assess non-emulsified LEO, Brozyna et al. also used a modified antibiofilm dressing’s activity measurement (ADAM) method. This is a peer reviewed method of assessing a dressing’s *in vitro* activity against biofilm-related wound infections (Junka et al., 2017). Based on the results of microdilution assays, three different clinical strains for each EO were selected and examined. To provide other research teams with the possibility of performance of this analysis, reference staphylococcal strains were also included. As a substance of proven antimicrobial activity, liquid phases of 96% (v/v) ethanol were applied (as controls of test usability). The concentration of EOs released from biocellulose discs was 65.8%. All EOs displayed an ability to eradicate biofilms (from 27% up to 92%).

Budzynska et al. (2011) colonised surfaces with bacterial strain tested and then incubated it with the LEO in a 96 well tissue culture microplate, and the activity was also measured with a TTC assay. These results were then further quantified through CFU determination.

A time-dependent eradication of biofilms assay was also performed and expressed as a minimal biofilm eradication concentration (MBEC), using concentrations ranging from their determined MIC to eight x MIC. The concentration of oil causing a 50%–90% reduction in biomass was recorded as the MBEC50 and MBEC90, and this was evaluated by the MTT reduction assay. Through this, it was determined that LEO required a rather high concentration (4 – 8 x MIC) to reach MBEC90.

Unlike Brozyna et al. (2021), Budzynska et al. (2011) evaluated the viability of bacterial membranes treated with essential oils with the LIVE/DEAD BacLight Bacterial Viability kit, photographing samples with a Hamamatsu digital camera. The study ascertained that while LEO had some antibiofilm potency, however other oils, such as Tea-Tree oil (TTO) and Melissa essential oil (MEO) had stronger anti-biofilm activity.

Thus, both studies displayed that LEO has antibiofilm potency against *S. aureus*. However, the exact efficacy recorded varies between these two studies, likely due to the different methodology, strains and LEO type.

#### 4.1.5 LEO in vapour form

Overall it seems LEO vapours have no to negligible effects on the growth of *S. aureus* strains. This result appears to be consistent across most studies which used similar variations of vapour disc diffusion and was also observed in the study which used a glass cylinder containing LEO. Brozyna et al. (2021) differed in methodology as they attempted to evaluate LEO vapour effectiveness against *S. aureus* biofilms using the antibiofilm activity of volatile compounds assay (AntiBioVol). Despite this difference in methodology, they also found LEO vapours to exhibit no antibiofilm activity. Voravuthikunchai et al. (2012) was the only study which demonstrated that LEO infused in the air had some antibacterial effects. However this effect could be explained as plates were left exposed to an air conditioning system for hours, which may have helped LEO to exhibit its antimicrobial effect. The other studies which tested LEO vapour antimicrobial activity conducted their experiments within a closed Petri dish as opposed to a system exposed to air conditioning.

### 4.2 LEO used synergistically with other agents

Overall LEO showed promising antibacterial synergism with other agents against *S. aureus*. Various substances were tested in conjunction with LEO, including Hap, antibiotics, octenidine, rhamnolipids and other EOs. Three studies showed LEO worked synergistically with antibiotics to increase their antibiotic effect. Ribeiro et al. (2020) demonstrated that LEO alone at sub-MIC concentrations exhibited minimal effects on *S. aureus*. However, when used in conjunction with penicillin, it indirectly caused the penicillin to increase its effectiveness 64 fold, restoring penicillin sensitivity to resistant strains to levels similar to sensitive strains. Both Adaszyńska-Skwirzyńska et al. (2020) and Cui et al. (2021) also reported a synergistic effect when LEO was combined with gentamycin against MRSA and MSSA. Three other studies combined LEO with substances other than antibiotics. Roller et al. (2009) combined LEO from different lavender species, and discovered that necrodane-rich LEO could produce larger inhibition zones against MSSA and MRSA. Haba et al. (2014) discovered that while LEO alone initially had no antibacterial effect against *S. aureus*, when emulsified with rhamnolipids, it managed to produce a zone of inhibition. Conversely, Di Vito et al. (2021) determined that there was no antibacterial effect against MRSA and MSSA when LEO was combined with hydrolates. Therefore LEO is a promising synergistic agent for antibiotics, especially gentamycin. LEO also has potential to work synergistically with other agents, and this is a prime area for further investigation, as there have only been three studies so far testing this area.

### 4.3 Strengths and limitations of this study

This systematic review has many strengths. As many studies have investigated a range of EOs against multiple bacteria species in their paper, the results regarding the actions of LEO against *S. aureus* are often not emphasised. Thus, by extracting this data, these efforts can be acknowledged, and the data can be used constructively to compare with other LEO results. Additionally, by collating and comparing many different types of LEO studies, it becomes easier to overview the types of studies other researchers have considered. In doing this, it is easier to observe which methods provide reliable and consistent results or whether novel methods should be considered instead. The inclusion of different LEO studies also allows us to observe any promising novel methods of using LEO, such as various vaporisation methods and possible synergistic combinations with other agents.

This review also has some limitations. Only four databases were searched. Additionally, not all papers had full text access. Only papers written in English were included, which may have limited the range of papers available, especially when EO treatments are of interest to other cultures. There are also no quality assessment tools publicly available to evaluate such variety *in vitro* studies. While examining a large variety of studies allows us to observe how researchers have contemplating using LEO, it also means results are difficult to compare.

## 5 Conclusion

Overall, LEO appears to have antimicrobial effect on some strains of *S. aureus* and MRSA. While multiple studies have observed an antimicrobial effect on *S. aureus* when LEO is used alone, the ranges and circumstances of its effectiveness varies, with some studies showing negligible effectiveness and others showing significant effectiveness. Therefore, the exact parameters of when LEO on its own exhibits and antimicrobial effect appears to vary. This range of results was likely attributed to the variety of study methods used across different papers. LEO vapours appear to have negligible effects on *S. aureus* and MRSA. LEO also appears to work synergistically with other antimicrobial agents, such as Hap, octenidine, other essential oils and other antibiotics. It is recommended that future research standardises LEO studies to allow for an easier comparison of results and the formulation of a decisive conclusion. Additionally, it may be of interest to further investigate compounds demonstrating synergistic action with LEO or to test other possible agents with LEO for synergistic activity.

## Data Availability

The original contributions presented in the study are included in the article/Supplementary material, further inquiries can be directed to the corresponding author.
